# Resilient Clinical Trial Infrastructure in Response to the COVID-19 Pandemic: Lessons Learned from the TOGETHER Randomized Platform Clinical Trial

**DOI:** 10.4269/ajtmh.21-1202

**Published:** 2022-01-07

**Authors:** Jamie I. Forrest, Angeli Rawat, Felipe Duailibe, Christina M. Guo, Sheila Sprague, Paula McKay, Gilmar Reis, Edward J. Mills

**Affiliations:** ^1^ Platform Life Sciences, Vancouver, Canada;; ^2^ University of British Columbia, Vancouver, Canada;; ^3^ University of Aberdeen, Aberdeen, Scotland;; ^4^ McMaster University, Hamilton, Canada;; ^5^ CardResearch, Belo Horizonte, Brazil;; ^6^ Pontifica Católica Universidade de Minas Gerais, Belo Horizonte, Brazil

## Abstract

In response to the COVID-19 pandemic, clinical research groups across the world developed trial protocols to evaluate the safety and efficacy of treatments for COVID-19. Despite this initial enthusiasm, only a small portion of these protocols were implemented. Of those implemented, a fraction successfully recruited their target sample size to analyze and disseminate findings. More than a year and a half into the COVID-19 pandemic, only a few clinical trials evaluating treatments for COVID-19 have generated new evidence. Productive randomized platform clinical trials evaluating COVID-19 treatments may attribute their success to intentional investments in developing resilient clinical trial infrastructures. Health system resiliency discourse provides a conceptual framework for characterizing attributes for withstanding shocks. This framework may also be useful for contextualizing the attributes of productive clinical trials evaluating COVID-19 therapies. We characterize the successful attributes and lessons learned in developing the TOGETHER Trial infrastructure using a health system resiliency framework. This framework may be considered by clinical trialists aiming to build resilient trial infrastructures capable of responding rapidly and efficiently to global health threats.

In 2020, clinical trial researchers developed protocols to evaluate the safety and effectiveness of potential treatments for COVID-19. Despite more than 3,000 trials registered (as of November 2021), few have generated findings, with the exception of smaller randomized controlled trials.^1^ Platform trials (e.g., SOLIDARITY,^2^ RECOVERY,^3^^–^^5^ Platform Randomised trial of INterventions against COVID-19 In older peoPLE [PRINCIPLE],^6^^–^^8^ REMAP-CAP,^9^^,^^10^ and TOGETHER,^11^^,^^12^) hold the most promise for the perpetual evaluation of COVID-19 therapeutics. The PRINCIPLE and TOGETHER Trials were similarly successful in recruiting patients and generating evidence, as compared and contrasted in Table 1. Although many similarities exist between these two trials, both of which focused on outpatient high-risk populations in community settings, the settings were different. The TOGETHER Trial took place in one state of a middle-income country (Brazil) with a more fragmented health system compared with the PRINCIPLE Trial, which took place within a well-integrated health system with a national health insurance scheme. The successes of these trials may be attributed to investments in a resilient clinical trial infrastructure. A clinical trial infrastructure (defined as the human, material, and knowledge networks that form a responsive implementation of productive trial protocols) must be resilient to threats (e.g., COVID-19), and is essential when barriers to sustainable funding are common (academic and public sectors).^13^

**Table 1:** Comparing and contrasting the TOGETHER and PRINCIPLE trials

Characteristic	TOGETHER trial	PRINCIPLE trial
Location	Brazilian state of Minas Gerais	United Kingdom
Patient population	Community setting, patients are at least 18 years of age, have a positive antigen test for severe acute respiratory syndrome coronavirus 2, and have an indication for high risk of disease severity, including comorbidities, older age, or high body mass index	Community setting, patients are 50-year-olds at high risk of complications (with comorbidities) or 65-year-olds or older who were unwell for up to 14 days with suspected COVID-19
Date recruitment began	June 2020	April 2, 2020
No. of participants recruited (November 2021)	3,800	7,833
Types of recruitment	Patients presenting to an outpatient clinic setting with clinical criteria for presumptive diagnosis of COVID-19 who met the eligibility criteria were invited to participate.	Twenty-five percent of participants were from 200 general practices; 75% were from online self-referral.
Strategies to reduce person-to-person contact	WhatsApp messaging and video for recruitment, communication with trial coordinators, and monitoring of participants at 13 clinical research sites; hotline	Online self-referral; participants received trial therapeutics via courier
No. of interventions	7	6
Therapeutics trialed vs. placebo or standard of care	Doxazosin, fluvoxamine, hydroxychloroquine, ivermectin, lopinavir/ritonavir, metformin, peginterferon lambda	Azithromycin, budesonide (inhaled), colchicine, doxycycline, favipiravir, ivermectin
Findings with promise for potential therapeutic value	Fluvoxamine reduced the chance of COVID-19-related hospitalization.	Inhaled budesonide improved time to recovery, with the potential to reduce hospital admissions or deaths.

PRINCIPLE = Platform Randomised trial of INterventions against COVID-19 In older peoPLE.

Health system resiliency provides a conceptual framework to evaluate and guide reforms during and after shocks (e.g., pandemics, natural disasters, political turmoil).^14^ The framework considers five domains: 1) *aware* (apply the best-known emerging evidence to inform decision making), 2) *diverse* (provide multiple pathways for patients and providers with various skills), 3) *self-regulating* (identify and re-allocate resources as needed), 4) *integrated* (draw efficiently on resources and expertise from other sectors as needed), and 5) *adaptive* (be able to change in response to new information).

This framework may be useful in characterizing productive clinical trial networks and infrastructure in responding to threats such as COVID-19. The TOGETHER Trial is an adaptive platform randomized clinical trial to evaluate re-purposed therapies for the treatment of early-diagnosed COVID-19 in the Minas Gerais state of Brazil.^12^ The ongoing trial (as of November 2021) has enrolled more than 3,800 patients and has published findings on the safety and effectiveness of treatment of early-diagnosed outpatient clinical management of COVID-19 with lopinavir/ritonavir or hydroxychloroquine,^11^ and fluvoxamine,^12^ with analyses of metformin, ivermectin, doxazosin, and peginterferon lambda pending (Figure 1). The TOGETHER Trial consortium is a network of investigators affiliated with academic institutions in Canada, Brazil, and the United States, in partnership with contracted private companies providing data management and analytical support.

**Figure 1.**
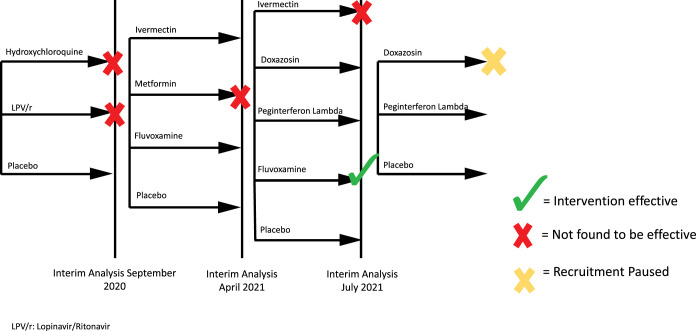
TOGETHER trial overview. LPV/r = lopinavir/ritonavir.

We describe the attributes of the TOGETHER Trial within the framework of health system resiliency. We identified four attributes of the TOGETHER Trial related to clinical trial infrastructure resiliency: 1) participants and partners; 2) knowledge, communication and evidence generation; 3) health system capacity and resourcing; and 4) governance, administration, and financing. By documenting these lessons learned, we demonstrate the utility of this framework as it applies to the development of a clinical trial infrastructure responsive to emergent global health threats (Figure 2).

**Figure 2.**
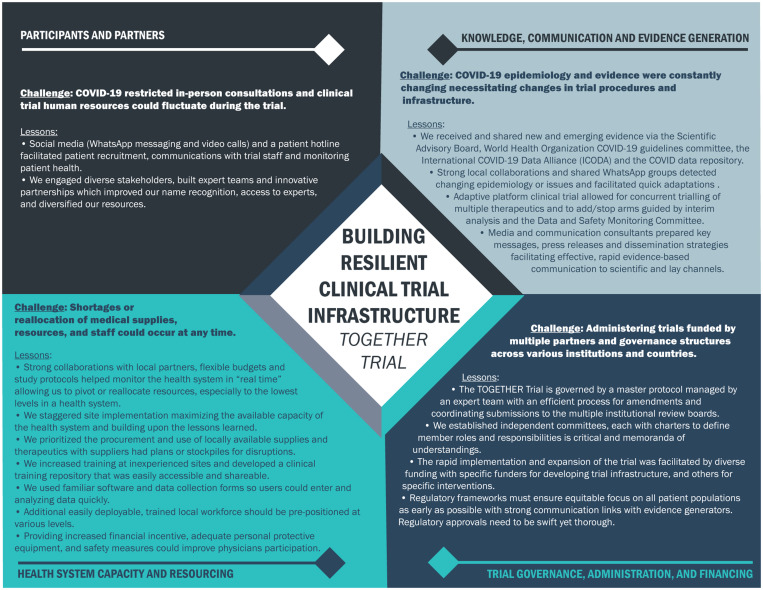
Summary of lessons learned.

*Participants and partners* refers to patient engagement and partnerships across the trial. In-person participant recruitment and monitoring were limited by the infectiousness of COVID-19 and the rapidly changing government lockdown scenarios limiting participants’ mobility. We used social media (WhatsApp, the most popular messaging app in Brazil) to recruit patients and connect them with trial coordinators, and to remind clinicians to follow up with patients and monitor patients (including adverse events) via video calls in lieu of in-person monitoring. Participants could also call a hotline to access clinicians and trial staff quickly.

Fluctuations in clinical trial human resources during a pandemic required rapid responses and innovative partnerships. We engaged experts and diverse stakeholders, including academics, pharmacists, clinicians, and statisticians, and experts in methodology, knowledge translation, guideline development, and ethics management. We expanded traditional partnerships and collaborations to new partners and other clinical trials, which improved our name recognition and access to experts, and diversified our resources.

*Knowledge, communication, and evidence generation* refers to how knowledge was generated, used, communicated, and disseminated. The ever-changing epidemiology and evidence related to COVID-19 challenged resilient clinical trial infrastructures. We had confidence in the evidence we received via our scientific advisory board, which reviewed new and emerging evidence regularly, and by having links with the WHO COVID-19 guidelines committee. Quick data sharing locally was facilitated by strong public-sector partnerships. We learned quickly from other evidence generators globally and shared our findings via the International COVID-19 Data Alliance and the COVID data repository.

With timeliness being critical in building a resilient trial infrastructure, we detected problems or changes early, shared information, and adapted quickly (e.g., shifting recruitment and clinical capacity as incidence rates changed). Strong collaborations with local partners monitored changing epidemiology in “real time” and facilitated quick responses. Shared WhatsApp groups within the steering committee and for each clinic allowed discussions to solve problems and identify issues early. Similar time zones facilitated knowledge sharing, and a designated interpreter ensured effective communication across languages. In collaborations with vastly different time zones, designated trial liaisons could function as on-call personnel responding to urgent requests outside of business hours.

One advantage of the adaptive platform clinical trial was that we trialed multiple investigational products simultaneously with the capacity to stop trial arms as evidence emerged of low or no efficacy, or to add potential therapeutics. Conducting interim analyses allowed us to detect changes at predetermined time points during the trial. The Data and Safety Monitoring Committee was flexible and advised rapidly on any adaptations (e.g., when to drop or add arms). Misinformation of study results and/or the politicization of therapeutics under investigation could impact trial participation and evidence uptake. For example, some patients refused participation, knowing they may be randomized to treatment with hydroxychloroquine—a politicized treatment of COVID-19 in Brazil.^15^ Having designated media and communication consultants who scanned online spaces constantly ensured our evidence was represented correctly. They also prepared key messages and dissemination strategies facilitating effective, evidence-based communication to scientific and lay channels. Releasing scientific evidence via press releases allowed for rapid evidence dissemination to meet the urgency and high demand for knowledge.

A major consideration for building resilience is the fluctuating *health system capacity and resources* available for clinical trials. Shortages or reallocation of medical supplies, resources, and staff could occur at any time. Strong collaborations with local partners monitored the health system in real time, allowing us to pivot or reallocate resources accordingly. Flexible budgets facilitated quick resource reallocation to all sectors of the health system participating in the trial. To accommodate changing human resource needs, an extra easily deployable, trained local workforce should be pre-positioned at various levels. All staff are provided with ongoing training, support, and supervision so they are current with procedures and protocols, further ensuring versatility in their job functions.

Supply chains could be disrupted and import channels could be limited, potentially leading to demand-driven price increases and limited or unavailable supplies. We first implemented in sites experienced with clinical trials and staggered site implementation thereafter to ensure we met the available capacity of the health system and built upon our lessons learned. We prioritized the procurement and use of locally available supplies and therapeutics or those procured from countries with no transfer or travel restrictions between them and Brazil. We ensured the suppliers had plans or stockpiles for disruptions. We did not anticipate the high costs for complex laboratory testing resulting from a lack of supply globally, and we recommend flexible study protocols and budgets to accommodate shocks in pricing.

Engaging diverse clinical sites also meant having disparities in human resource capacities and clinical trial experience. We increased training at inexperienced sites and developed a clinical training repository that was easily accessible and shareable. Resilient referral networks were built, where participants were monitored frequently by physicians via WhatsApp video calls, and were linked quickly and referred to larger hospitals. By using familiar software and data collection forms, and ensuring users’ trainings were up-to-date, we avoided steep learning curves that prevent users from entering and analyzing data quickly. Physicians could be deterred from participating in trials when the disease transmission is novel or the populations are in high-risk areas. Providing an increased financial incentive, adequate personal protective equipment, and safety measures could improve participation.

*Trial governance, administration, and financing* must also be resilient. The primary attribute of a resilient platform trial is the execution of a master protocol, detailing trial procedures for the evaluation of multiple interventions to treat the same disease within a single overall trial structure.^16^ The TOGETHER Trial is governed by a master protocol that is flexible to trial adaptations, including rules for adding and dropping arms. The master protocol for the TOGETHER Trial was managed by an expert team affiliated with the sponsor institution and the institutional review board of record, which fostered an efficient process for amending the master protocol and coordinating submissions to the multiple institutional review boards that govern the trial.

Establishing independent committees, each with charters to define member roles and responsibilities, is critical. The TOGETHER Trial developed charters for the following committees: scientific steering, central trial coordination, local trial implementing, data and safety monitoring, and event adjudication. For example, the local implementing committee established memoranda of understandings with each clinical site, which facilitated the rapid expansion of the trial network throughout the state of Minas Gerais.

Diversifying funding is important for sustaining resilient clinical trial networks. The TOGETHER Trial is funded by private donors and philanthropic organizations that accommodated the unique limitations and scientific priorities of each party. We secured funding dedicated to developing clinical trial infrastructure, whereas other funders supported the trial activity implementation for specific interventions. Although patchwork funding from diverse sources has facilitated the rapid implementation and expansion of the TOGETHER Trial, a transition to more sustainable and long-term funders is essential to support the perpetual evaluation of potential interventions.

Evidence generated from such trials can only be as effective as the systems in place to uptake the evidence quickly. Early in the COVID-19 response, evidence generated from inpatient trials were prioritized. We missed an opportunity to generate concurrently evidence for outpatient populations. Although outpatients have a lower probability of developing severe illness, interventions could greatly reduce the need for health system resources.^17^ Therefore, we recommend regulatory frameworks ensure an equitable focus on all patient populations as early as possible, with strong communication links with evidence generators, and regulatory and implementing authorities. Regulatory approvals need to be swift yet thorough.

To our knowledge, this is the first documentation of a platform trial infrastructure in the COVID-19 era and the first to characterize attributes of the trial infrastructure within an analogous framework to health system resiliency. We identified four attributes for a resilient clinical trial infrastructure. Although this framework may be useful for other clinical trial networks aiming to build resilient infrastructures, the generalizability of the lessons from the TOGETHER Trial described herein are limited by the geographic, social, and political context of the Minas Gerais state of Brazil. The emergence of COVID-19 as a global health threat and the urgent clinical response it demanded highlight the critical need for clinical trialists to build resilient trial infrastructures. Lessons learned from the TOGETHER Trial may help other trial networks respond quickly or more efficiently to future threats and may have implications for other trials or interventions.
